# Recovery-Based Occluded Face Recognition by Identity-Guided Inpainting

**DOI:** 10.3390/s24020394

**Published:** 2024-01-09

**Authors:** Honglei Li, Yifan Zhang, Wenmin Wang, Shenyong Zhang, Shixiong Zhang

**Affiliations:** 1 School of Computer Science and Engineering, Macau University of Science and Technology, Macau, China; 2009853cia30001@student.must.edu.mo (H.L.); 3230002497@student.must.edu.mo (Y.Z.); 2009853gia30007@student.must.edu.mo (S.Z.); 2009853gia30002@student.must.edu.mo (S.Z.); 2 Chongqing College of Electronic Engineering, Chongqing 401331, China

**Keywords:** occluded face recognition, identity-guided inpainting, image synthesis, generative adversarial net (GAN)

## Abstract

Occlusion in facial photos poses a significant challenge for machine detection and recognition. Consequently, occluded face recognition for camera-captured images has emerged as a prominent and widely discussed topic in computer vision. The present standard face recognition methods have achieved remarkable performance in unoccluded face recognition but performed poorly when directly applied to occluded face datasets. The main reason lies in the absence of identity cues caused by occlusions. Therefore, a direct idea of recovering the occluded areas through an inpainting model has been proposed. However, existing inpainting models based on an encoder-decoder structure are limited in preserving inherent identity information. To solve the problem, we propose ID-Inpainter, an identity-guided face inpainting model, which preserves the identity information to the greatest extent through a more accurate identity sampling strategy and a GAN-like fusing network. We conduct recognition experiments on the occluded face photographs from the LFW, CFP-FP, and AgeDB-30 datasets, and the results indicate that our method achieves state-of-the-art performance in identity-preserving inpainting, and dramatically improves the accuracy of normal recognizers in occluded face recognition.

## 1. Introduction

In recent years, occluded face recognition has become a research hotspot in computer vision. Unlike unoccluded faces, occluded faces suffer from incomplete visual components and insufficient identity cues, which lead to degradation in recognition accuracy by normal recognizors [1,2,3,4]. Inspired by the recovery mechanism of the nervous system, researchers have proposed two types of approach, i.e., occlusion-robust and occlusion-recovery.

The occlusion-robust approach attempts to improve the robustness of recognizers on occluded faces by improving the “representation”. The latest work, FROM [5], proposed an end-to-end occluded face recognition model to learn the feature masks and deep occlusion-robust features simultaneously. However, compared with normal recognizers, it has weakened generalization ability over datasets with wide age and angle differences, such as the CFP-FP [6] and AgeDB-30 [7].

**Figure 1:**
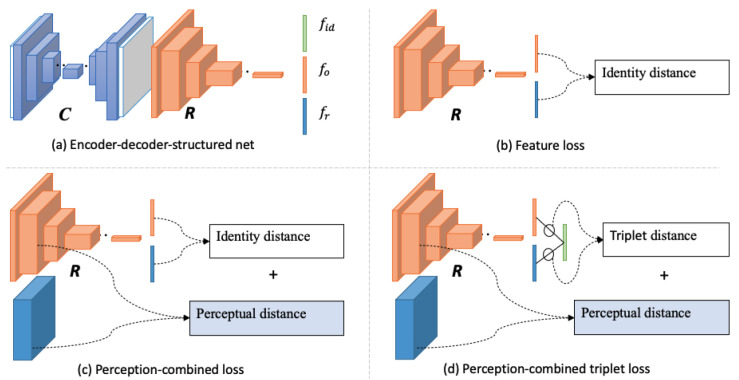
Encoder-decoder-structured identity-preserving inpainting networks with different identity training loss. C is an encoder-decoder-structured content inpainting network, and R is a pretrained recognizer. $f_{id}$, $f_{o}$, $f_{r}$ are identity-centered features, occlusion-recovered features, and real face features, respectively.

Unlike the occlusion-robust approach, the occlusion-recovery approach recovers the occluded regions before recognition. GAN-based inpainting methods [8,9] have remarkably improved realistic content generation. At the same time, identity-preserving inpainting models [10,11,12,13,14,15] have been demonstrated to be effective for occluded face recognition. These methods often adopt encoder-decoder-structured networks but with different identity loss during training, as Figure 1 shows. Dolhansky et al. [10] imported identity features to preserve identity information in eye regions by L2 feature loss, as Figure 1b shows. Inspired by the perceptual loss [11,12,16] used identity loss which combined perceptual items and identity feature items, as Figure 1c shows. The perceptual item is computed with semantic features from a low-level layer of the pretrained recognizer, while the identity feature item is from the output of the top-level layer. Ge et al. [15] proposed an identity-diversity loss that combines perceptual loss and identity-centered triplet loss to guide face recovery, which achieved state-of-the-art performance in identity preserving inpainting, as Figure 1d shows. Duan et al. [13] designed two-stage GAN models to deal with face completion and frontalization simultaneously. However, these methods are also limited by the challenge of preserving the inherent identity information against large occlusions. These methods often utilize incomplete datasets to learn the identity distribution with the supervision of identity and reconstruction loss functions, which makes the learned distribution deviate from its real one. Then, the decoder generates a new face from sampling the biased identity space, further enhancing the identity offset of the generated image.

**Figure 2:**
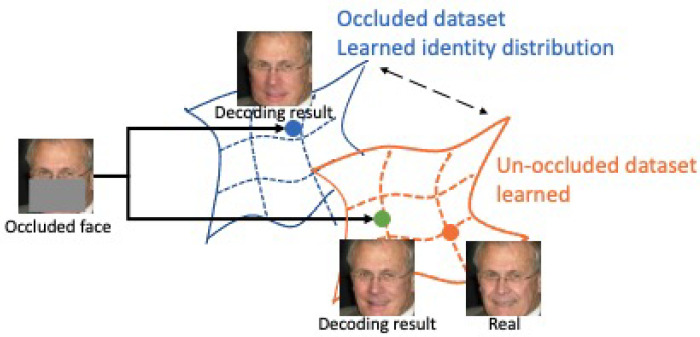
We get the recovered result closer to the ground truth by sampling from a closer distribution, which is learned with an unoccluded dataset.

This work uses a GAN-like identity-guided inpainting model to solve occluded face recognition. We refer to our method as ID-Inpainter for brevity. Instead of starting from a Gaussian distribution, our model samples from an identity distribution learned with an unoccluded dataset, which reaches closer to the real distribution than that with an occluded dataset. The difference is shown in Figure 2. Our ID-Inpainter consists of a content inpainting process and an identity fusing process. In the content inpainting process, we train a content inpainter to implement a coarse recovery with structure consistency. In the fusing process, we design a GAN-like identity fusor consisting of a series of adaptive identity fusion blocks (AIFBs) to fuse the identity and attribute features. Through the GAN-like fusor and specifically designed AIFBs, we achieve more efficient identity fusing and obtain better attribute-consistent inpainting results.

## 2. Related Work

### 2.1. Occluded Face Recognition

Face recognition is a computer vision task that recognizes the identity among multiple face images. It is closely related to feature extraction, classification [17], and detection [18] technology. As one of the most successful practical cases, face recognition has a long history of research which has extended to various application scenarios [15,19,20]. Traditional face models are designed for unoccluded face images (see, for example, [1,2]). When they are applied directly to occluded datasets, their accuracy drops dramatically. There are two main approaches to solving the problem: occlusion-robust and occlusion-recovery.

The occlusion-robust approach reduces the accuracy drop by improving the robustness of recognizers on occluded faces. One idea is to improve the “representation”. Refs. [21,22,23] report various kinds of representation methods for facial features. The latest work called FROM [5] is an end-to-end occluded face recognition model to learn the feature masks and deep occlusion-robust features simultaneously and achieved the SOTA result on the occluded LFW dataset.

Unlike the occlusion-robust approach, the occlusion-recovery approach recovers the occluded facial regions and then performs recognition on the recovered faces. Ge et al. [15] proposed an identity-diversity inpainting network to facilitate occluded face recognition. It improved the recovery step by integrating GAN with a novel CNN network, which used identity-centered features as supervision to enable the inpainted faces to cluster towards their identity centers. In [14], occlusions were removed with a CNN-based deep inpainting network. However, these methods are also limited by the challenge of preserving the inherent identity information against large occlusions. The core reason lies in the insufficient transformation of identity information. So, if we can improve the identity information transformation in the inpainting phase, we will further improve the performance of occluded face recognition.

### 2.2. Identity-Preserving Face Inpainting

A simple approach for face inpainting is to borrow general deep learning inpainting methods directly, which are good at rebuilding the overall structure of the face. For example, generative inpainting methods [9,24] involve the design of attention layers to improve the global structure consistency and fidelity and have performed well in face inpainting. Although these methods have been shown to maintain the consistency of facial structure, they showed limited improvement in occluded face recognition. So, some researchers have turned their attention to identity-preserving face inpainting.

Identity-preserving face inpainting attempts to perceive the identity information from the uncorrupted region. Some attempts, e.g., [14,15,25], imported identity loss to solve the problem and were demonstrated to be effective for occluded face recognition, but not significantly. For example, Ge et al. [15] proposed an identity-preserving face completion model that combined a CNN network and a third recognizer player to complete identity-diversity inpainting. It was designed explicitly for occluded face recognition but failed to improve performance on large-size occlusions. The main reason is that the traditional encoder-decoder network trained on occluded datasets can not build real identity space, leading to a prominent identity offset in the inpainting process. Li et al. [26] creatively combined a general inpainting network with AAD-generator [27] to solve identity-guided inpainting tasks, regenerating missing content from a pretrained identity distribution. However, there is still a certain distance in style and structure between the generated face and the ground truth face. Although an additional Poisson blending module is used to repair the style difference, the structure bias cannot be erased.

### 2.3. Normalization Layers

GAN is powerful in generating photo-realistic results based on distribution sampling. There have been broad investigations of the normalization layers [26,27,28,29] in GANs to improve the prediction performance. Among them, spatially adaptive denormalization (SPADE) [28] and adaptive attentional denormalization(AAD) [30] are related to our AIFB. By relying on the prelearned identity distribution and AIFBs, our method can effectively fuse the identity information into the missing area and maintain a high degree of structural consistency.

## 3. Proposed Method

**Figure 3:**
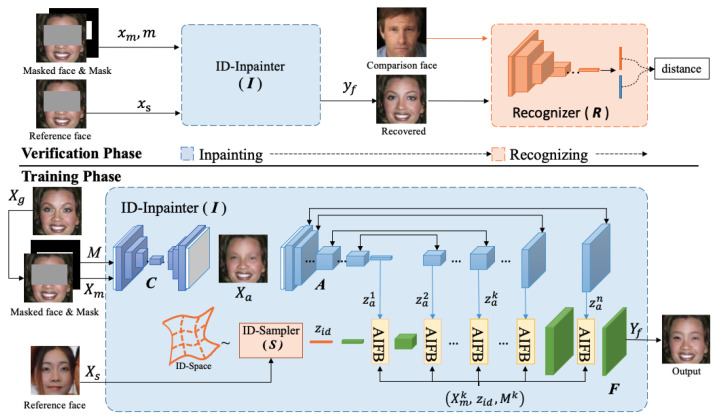
The overall pipeline of our approach. It is divided into verification and training phases. The verification phase consists of two modules: ID-Inpainter ***I*** and recognizer ***R***. ID-Inpainter ***I*** consists of three sub-networks, i.e., content inpainter ***C***, identity sampler ***S***, and identity fusor ***F***. In the training phase, ground truth faces $X_{g}$, occlusion masks *M*, and reference images $X_{s}$ are put into ***I*** to train an identity-guided inpainting model. In the verification phase, the masked face is used as the reference face to implement identity-preserving inpainting. Finally, the inpainted result is recognized by a normal recognizer ***R***.

For occlusion-recovery face recognition, the recovery model inpaints the occluded face to meet structure consistency and identity preservation. Instead of using a traditional encoder-decoder generator, we utilize a GAN-like identity-guided face inpainting network for the inpainting, as shown in Figure 3. Our method consists of two phases: the verification phase and the training phase. In the training phase, we use occlusion-free faces as the reference image while adopting the masked face as the reference in the verification phase.

### 3.1. Problem Definition

Given a ground truth face $x_{g}$ and its occluded version $x_{m}$, our goal is to inpaint the occluded image with structure-consistent and identity-preserving content to make it easier to be recognized by normal recognizers. During the inpainting process, we use a mask *M* to indicate the occluded areas, and a reference face $x_{s}$ to guide the identity-preserving inpainting. As Figure 3 shows, our ID-Inpainter I consists of a content inpainter C, an identity sampler S, an attribute extractor A, and an identity fusor F. In the training phase, we obtain content recovered outputs $X_{a}$ by $X_{a}=C\left({\tilde{X}}_{m},M\right)$, the identity embeddings $z_{id}$ by $z_{id}=S\left(X_{s}\right)$, and the multi-scaled attribute embeddings $z_{a}$ by $z_{a}=A\left(X_{a}\right)$. Then, the $\left\{z_{a},z_{id},M,X_{a}\right\}$ are delivered to the identity fusor F to obtain the $Y_{f}$. According to our goal, we need to maintain the structure consistency between $Y_{f}$ and $X_{g}$, while maximizing the identity similarity between $Y_{f}$ and $X_{g}$. The process can be formulated as

(1a) $$\begin{array}{cc} Y_{f} & =F\left(A\left(C\left(X_{m},M\right)\right),S\left(X_{s}\right),X_{m},M\right) \end{array}$$

(1b) $$\begin{array}{cc} A\left(X_{g}\right) & ≐A\left(Y_{f}\right) \end{array}$$

(1c) $$\begin{array}{cc} S\left(X_{g}\right) & ≐S\left(Y_{f}\right) \end{array}$$

where ≐ means “equivalence” in some metric.

However, the $X_{g}$ is unknown in the verification phase. Assuming that we can find an alternative $X_{s}$ which is very similar in identity to $X_{g}$, we could update Equation (1c) as

(2) $$S\left(X_{s}\right)≐S\left(Y_{f}\right)\;if\;S\left(X_{s}\right)≐S\left(X_{g}\right).$$

Now, the questions are how to find the very similar $X_{s}$ and how to transmit more identity information to the fused result $Y_{f}$ with high structural consistency.

### 3.2. Identity-Guided Inpainting

To keep structural consistency, we implement the content inpainting module *C* by rebuilding the network of DeepFill [8] to meet the input size of 112×112. Inspired by SPADE [31] and SwapInpaint [26], we utilize a GAN-like identity fusor to deal with identity-guided inpainting. To fuse more identity information in the recovered result, we replace the Gaussian space of the traditional GAN with the identity space and adopt a recognizer trained with an occlusion-free dataset as the identity sampler. Here, we use an Arcface built on ResNet50-IR [2] with a feature dimension of 256, with unoccluded CASIA-WebFace [32]. The identity fusor contains a series of modulation blocks with upsampling layers. Assuming that we define the *k*-th modulation block as $f^{k}$, the *k*-th fused output $Y_{f}^{k}$ is produced by

(3) $$Y_{f}^{k}=f^{k}\left(Y_{f↑}^{k-1};z_{a}^{k},z_{id},X_{m},M\right),k\in \left\{1,2,\ldots ,7\right\}$$

where $Y_{f↑}^{k-1}$ is the upsampled result of $Y_{f}^{k-1}$ to match the *k*-th level. $Y_{f}^{0}$ is the output of a 2× deconvolution on the $z_{id}$. Similar to SwapInpaint [26], the attribute extractor ***A*** is a UNet A to convert the $X_{a}$ into multi-scaled attributes $z_{a}$.

**Figure 4:**
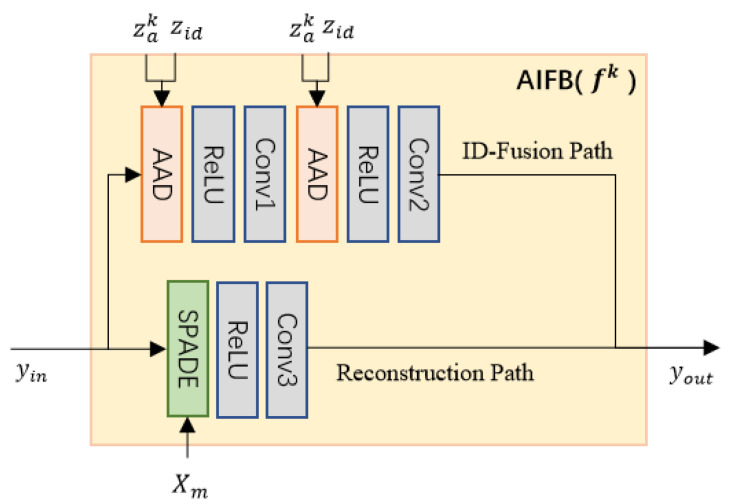
The structure of *k*-th AIFB. Each block consists of an ID-fusion path and a reconstruction path.

To decrease the structure and style differences in inpainting scenarios, we improve the AAD [27] to the attribute and identity fusing block (AIFB), which combines SPADE and AAD into a residual block. As Figure 4 illustrates, each AIFB is divided into ID-fusion and reconstruction paths. The ID-fusion path consists of two AADs responsible for the fusion of $z_{id}$ and $z_{a}^{k}$, while the reconstruction path utilizes a SPADE module to rebuild the unoccluded region of the input image $X_{a}$.

It may be noted that, according to Equation (2), in the verification phase, we need to find a reference image $x_{s}$, which should be as close to the ground truth $x_{g}$ as possible in identity space. From the quantitative comparisons, we find that some normal recognizers still maintain certain generalizations on occluded images; for example, the ArcFace [2] can reach a verification accuracy of 85.28% on 64 random occluded LFW [33]. Therefore, it is reasonable to infer that various occluded versions of the same image still have cohesive properties in identity space and can be used directly as the reference image in the verification phase.

### 3.3. Training Process

For the content inpainter C, the training process is the same as DeepFill [8]. For the identity fusor, which we call ID-Fuser for short, we train the attribute extractor ***A*** and the fusor ***F*** jointly. The training set is $\left\{X_{g},X_{s},M\right\}$. $X_{s}$ is randomly set to be the same or different from the $X_{g}$. As for the loss function, we use a reconstruction loss to train the attribute extractor and the reconstruction path when the reference images are the same as the ground truth images, i.e.,

(4) $$L_{rec}=\left\{\begin{array}{cc} \frac{1}{2}{∥Y_{f}-X_{g}∥}_{2}^{2} & if\;X_{g}=X_{s};\\ 0 & otherwise. \end{array}\right.$$

For the ID-Fusion path, we use l2 loss between the attribute embeddings to maintain the attribute consistency, which is formulated as

(5) $$L_{att}=\frac{1}{2}{∥A\left(Y_{f}\right)-A\left(X_{g}\right)∥}_{2}^{2}.$$

At the same time, an identity loss is used to fuse the identity information of the reference face. It is computed as

(6) $$L_{id}=1-cos\left(S\left(Y_{f}\right),S\left(X_{s}\right)\right),$$

where $cos\left(\cdot ,\cdot \right)$ represents the cosine similarity of two embeddings.

Furthermore, we need a multi-scale GAN loss [27] to make the result realistic. Then, the final loss is formulated as

(7) $$L=\lambda_{1}L_{rec}+\lambda_{2}L_{att}+\lambda_{3}L_{id}+L_{gan}$$

## 4. Experiment Results

### 4.1. Experiment Settings

We take CelebA [34], which is a large-scale face attributes dataset with more than 200 K celebrity camera-captured photos as the training datasets for all the comparison models, while LFW [33], CFP-FP [6], AgeDB-30 [7], and FaceScub [35] are used as the test datasets. The faces are aligned for all datasets and cropped to 112×112 resolution. The occluded versions are synthesized as in [9]. We extract 2 k images for validation; the others are used for training. For the loss weights, which are set by default as $\lambda_{1}=\lambda_{2}=10$, $\lambda_{3}=5$, we gradually increase the value of $\lambda_{3}$ during training from 5 to 10. When training, the ratio of the same to cross-identity paires is set to 1:1. All models use the Adam optimizer with the beta parameter set as $\left[0.1,0.999\right]$, and the learning rate as $10^{-4}$. ID-Fuser is trained for 100,000 iterations in total, while the content inpaintor and other inpainting models for comparison are all trained for 500,000 iterations. We implement our model with PyTorch 1.7.1 on a single NVIDIA V100 with a batch size of 16.

### 4.2. Comparison Experiments

#### 4.2.1. Face Inpainting

**Figure 5:**
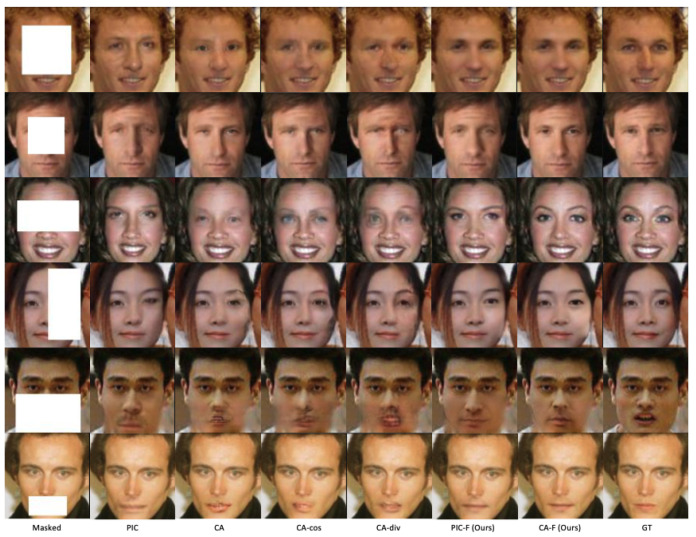
Inpainting results generated by different models. In each row, from left to right, they are the masked face, inpainting result by PIC [9], CA [8], CA with cosine identity loss (CA-cos), and CA with central-diversity loss [15] (CA-div), ID-Inpainter on PIC (PIC-F), ID-Inpainter on CA (CA-F), and the ground truth (GT).

We compare the proposed ID-Inpainter based on the content inpainter of PIC and CA with PIC [9], CA [8], CA with cosine identity loss (the same as ExGAN [10]), and CA with central-diversity loss (the same as ID-GAN [15]) on face inpainting in Figure 5. It can be seen that our ID-Inpainters achieve better visual quality than the others. Moreover, our models achieve better inpainting quality and higher identity similarity, as shown in Table 1.

#### 4.2.2. Face Recognition

We evaluate the recognition performance of PIC [9], CA [8], CA-cos, CA-div, and ID-Inpainter on the occluded LFW dataset. All experiments are performed on the random block of 48×48, the random block of 64×64, and the random-part occlusions. The random block is implemented by placing block occlusion at a random location, including the mouth, left eye, right eye, nose, left face, right face, upper face, two eyes, and lower face. The results in Table 2 demonstrate several essential observations. First, structure consistency plays a role in improving the recognition accuracy. For content inpainting, CA performs better than VAE-based PIC. Second, the area of missing blocks has a significant influence on recognition. Lastly, compared with CAs built with an encoder-decoder network, our ID-Inpainter achieves a higher score for occluded face recognition.

### 4.3. Analysis of the Framework

#### 4.3.1. Effects of Different Occluded Areas

From existing research, we know that different occluded areas affect the recognition differently. In this experiment, we quantitatively evaluate the influence on the LFW dataset. We explore occlusion types of the left eye, right eye, mouth, nose, two eyes, left face, right face, upper face, and lower face. The results in Table 3 show that occluded areas have the same effects on our method. For example, our method achieves high accuracy in the mouth area but suffers from sharp degradation in the eyes areas. At the same time, it demonstrates that our ID-Inpainter contributes to an accuracy increase in every part.

#### 4.3.2. AIFBs

**Figure 6:**
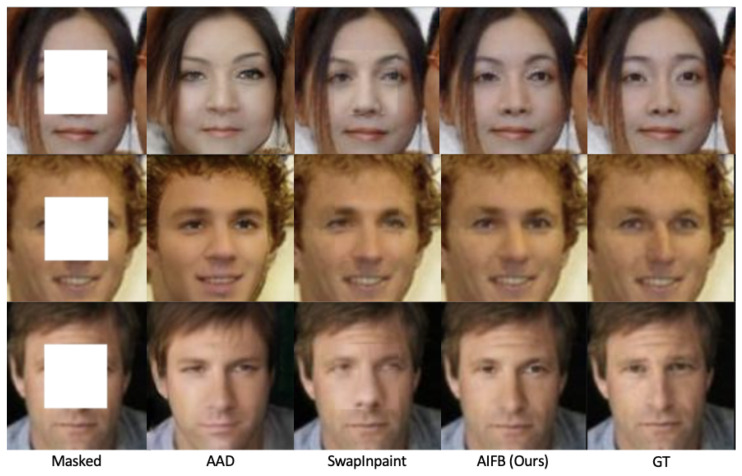
Inpainting results from different modulation modules.

We propose an AIFB to shorten the distance between the inpainted result and the ground truth in style and structure. Here, we compare our results with the AAD-Generator [27], which uses the ID-fusion path only, and SwapInpaint [26] without post-processing. As shown in Figure 6, AAD-Generator and SwapInpaint effectively transfer identity information but can not keep the unoccluded region unchanged.

#### 4.3.3. Identity Space

**Figure 7:**
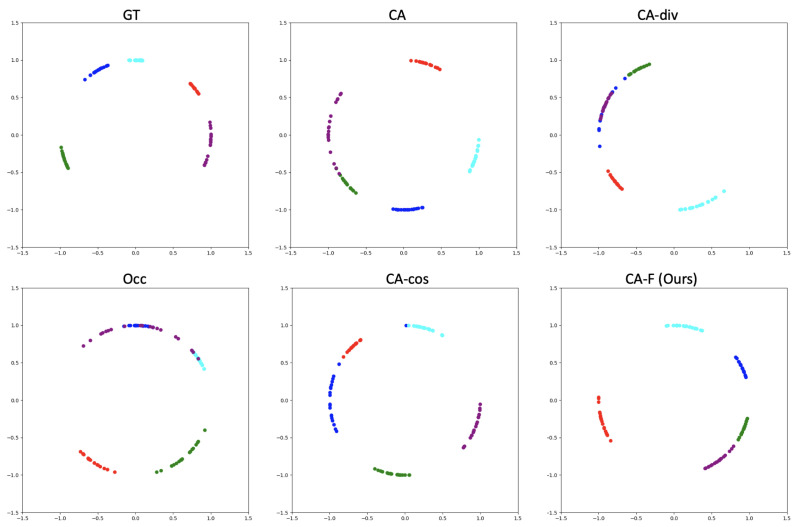
Visualization of feature distributions by converting 256D to 2D with t-SNE [36] and following normalization. Different markers with color represent different classes. Zoomed in for better view.

To explore the influence of ID-Inpainter on occluded face recognition, we compare the identity distributions among four test datasets, i.e., the ground truth (GT), occluded (Occ.), CA, and ID-Inpainter. Five classes with 20 samples for each in FaceScub [35] are randomly picked and are projected to a 256D identity space by ArcFace [2]. After that, we use t-SNE [36] to reduce the dimensions from 256 to 2 and visualize them after normalization, as in Figure 7. The highly aggregated features on ground truth are dispersed due to occlusions. CAs mitigate some dispersion but still fail to tell these classes apart. However, ID-Inpainter makes the features more cohesive based on CA and distinguishes these classes with more apparent margins.

#### 4.3.4. More test datasets

We report the verification experiment results for LFW-112, CFP-112, and AgeDB-112 in Table 4. Each dataset is compared with FROM [5], ArcFace [2], and our ID-Inpainter on different occlusions. These results demonstrate that our approach still works for the test datasets that vary widely in age and angle.

## 5. Conclusions

We proposed ID-Inpainter, a new identity-guided face inpainting network for occluded face recognition. It achieves maximum identity preservation through a GAN-like fusing network. However, many challenges remain to be tackled when it is applied in real-world scenarios. For example, we can not use it directly in real occlusions. When we meet real occlusion datasets, such as RMFRD [37], Bus Violence [38], CrowdSim2 [39], etc., we must combine it with an automatic occlusion detector. At the same time, the existing face occlusion detectors do not always perform perfectly to obtain the occlusion masks, which may negatively impact the subsequent inpainting process. Most occlusion detectors are built on a segmentation model and trained with synthesized datasets, which perform poorly in detecting real images. Appropriate improvements in datasets and algorithm strategies can significantly improve the accuracy of occlusive masks, thus ensuring recognition performance. For example, they could increase the proportion of real occluded images in the training dataset or improve the algorithm to obtain the occlusions indirectly based on detecting the face background. Another obvious challenge is the balance of structure consistency and identity preservation. A set of appropriate loss weight settings and the ratio setting of the same-identity pairs in the training dataset are needed to obtain optimal performance.

Combined with occlusion detectors, our model can play an essential role in various occluded face recognition scenarios, such as suspect retrieval, access verification, etc. In the future, we plan to extend our work to blind inpainting, which will rely little on the occlusion detector and is anticipated to be more effective when applied practically.

## Figures and Tables

**Table 1:** Quantitative performance on inpainting results. Arrows indicate whether larger is better or smaller is better, and bold indicates the optimal value.

Model	SSIM ↑	PSNR ↑	FID ↓	Identity ↑
PIC	0.8764	26.2543	3.6883	78.38
CA	0.8902	27.0059	3.5340	81.10
CA-cos	0.8898	27.2068	3.3807	81.76
CA-div	0.8876	27.0058	3.0075	81.46
PIC-F (Ours)	0.8844	27.0531	2.9969	82.76
CA-F (Ours)	**0.9091**	**28.8303**	**2.7254**	**85.55**

**Table 2:** Verification accuracy (%) of occlusion-recovery methods. Bold indicates the best value.

Mask	Occ.	PIC	PIC-F (Ours)	CA	CA-cos	CA-div	CA-F (Ours)
R-block (48)	95.03	96.30	**96.85**	96.31	97.39	97.24	**97.58**
R-block (64)	85.28	89.97	**93.08**	91.92	92.19	92.53	**94.13**
R-part	91.46	92.96	**95.03**	95.00	93.80	95.20	**96.65**

**Table 3:** Results for the effect of our ID-Inpainter with different recognizers. The results are measured by verification accuracy (%).

Data	Mouth	Left Eye	Right Eye	Nose	Left Face	Right Face	Upper Face	Two Eyes	Lower Face
**ArcFace (GT: 99.30)**									
Occluded.	98.58	97.40	98.16	95.03	93.34	95.86	83.76	89.20	92.03
Inpainted	99.36	98.92	98.85	98.93	98.13	98.05	91.75	94.22	93.50
Improvement	+0.8	+1.5	+0.7	+3.9	+4.8	+2.2	+8.0	+5.0	+1.5

**Table 4:** Results for LFW, CFP-FP, and AgeDB-30. The results are measured by verification accuracy (%). Bold indicates the best value.

Dataset	Occlusion	FROM	ArcFace	ID-Inpainter
LFW	R-block(48)	**98.43**	95.03	97.58
	R-block(64)	**97.15**	85.28	94.13
	R-part	**97.53**	91.46	96.65
CFP-FP	R-block(48)	55.58	83.43	**89.78**
	R-block(64)	54.12	69.45	**77.56**
	R-part	54.06	79.26	**84.40**
AgeDB-30	R-block(48)	51.85	79.90	**87.87**
	R-block(64)	51.62	67.71	**77.73**
	R-part	51.26	74.51	**84.03**

## Data Availability

Publicly available datasets were analyzed in this study. The processed datasets can be found here: https://github.com/icaoyu/ID-Inpainter.

## References

[1] Wang H., Wang Y., Zhou Z., Ji X., Gong D., Zhou J., Li Z., Liu W. Cosface: Large margin cosine loss for deep face recognition. Proceedings of the IEEE Conference on Computer Vision and Pattern Recognition.

[2] Deng J., Guo J., Xue N., Zafeiriou S. Arcface: Additive angular margin loss for deep face recognition. Proceedings of the IEEE/CVF Conference on Computer Vision and Pattern Recognition.

[3] Deng J., Guo J., Yang J., Lattas A., Zafeiriou S. Variational prototype learning for deep face recognition. Proceedings of the IEEE/CVF Conference on Computer Vision and Pattern Recognition.

[4] Huang Y., Wang Y., Tai Y., Liu X., Shen P., Li S., Li J., Huang F. Curricularface: Adaptive curriculum learning loss for deep face recognition. Proceedings of the IEEE/CVF Conference on Computer Vision and Pattern Recognition.

[5] Qiu H., Gong D., Li Z., Liu W., Tao D. (2021). End2End occluded face recognition by masking corrupted features. IEEE Trans. Pattern Anal. Mach. Intell..

[6] Sengupta S., Chen J.C., Castillo C., Patel V.M., Chellappa R., Jacobs D.W. (2016). Frontal to profile face verification in the wild. Proceedings of the 2016 IEEE Winter Conference on Applications of Computer Vision (WACV).

[7] Moschoglou S., Papaioannou A., Sagonas C., Deng J., Kotsia I., Zafeiriou S. Agedb: The first manually collected, in-the-wild age database. Proceedings of the IEEE Conference on Computer Vision and Pattern Recognition Workshops.

[8] Yu J., Lin Z., Yang J., Shen X., Lu X., Huang T.S. Free-form image inpainting with gated convolution. Proceedings of the IEEE/CVF International Conference on Computer Vision.

[9] Zheng C., Cham T.J., Cai J. Pluralistic image completion. Proceedings of the IEEE/CVF Conference on Computer Vision and Pattern Recognition.

[10] Dolhansky B., Ferrer C.C. Eye in-painting with exemplar generative adversarial networks. Proceedings of the IEEE Conference on Computer Vision and Pattern Recognition.

[11] Li C., Ge S., Hua Y., Liu H., Jin X. (2020). Occluded face recognition by identity-preserving inpainting. Cognitive Internet of Things: Frameworks, Tools and Applications.

[12] Duan Q., Zhang L. (2020). Look more into occlusion: Realistic face frontalization and recognition with boostgan. IEEE Trans. Neural Netw. Learn. Syst..

[13] Duan Q., Zhang L., Gao X. (2021). Simultaneous face completion and frontalization via mask guided two-stage GAN. IEEE Trans. Circuits Syst. Video Technol..

[14] Din N.U., Javed K., Bae S., Yi J. (2020). A novel GAN-based network for unmasking of masked face. IEEE Access.

[15] Ge S., Li C., Zhao S., Zeng D. (2020). Occluded face recognition in the wild by identity-diversity inpainting. IEEE Trans. Circuits Syst. Video Technol..

[16] Johnson J., Alahi A., Fei-Fei L. (2016). Perceptual losses for real-time style transfer and super-resolution. Computer Vision—ECCV 2016: 14th European Conference, Amsterdam, The Netherlands, 11–14 October 2016, Proceedings, Part II 14.

[17] Ullah A., Jami A., Aziz M.W., Naeem F., Ahmad S., Anwar M.S., Jing W. Deep Facial Expression Recognition of facial variations using fusion of feature extraction with classification in end to end model. Proceedings of the 2019 4th International Conference on Emerging Trends in Engineering, Sciences and Technology (ICEEST).

[18] Ahmad T., Ahmad S., Rahim A., Shah N. (2023). Development of a Novel Deep Convolutional Neural Network Model for Early Detection of Brain Stroke Using CT Scan Images. Recent Advancements in Multimedia Data Processing and Security: Issues, Challenges, and Techniques.

[19] Zhang T., Wiliem A., Yang S., Lovell B. (2018). Tv-gan: Generative adversarial network based thermal to visible face recognition. Proceedings of the 2018 International Conference on Biometrics (ICB).

[20] Afzal S., Ghani S., Hittawe M.M., Rashid S.F., Knio O.M., Hadwiger M., Hoteit I. (2023). Visualization and Visual Analytics Approaches for Image and Video Datasets: A Survey. ACM Trans. Interact. Intell. Syst..

[21] Qian J., Yang J., Zhang F., Lin Z. Robust low-rank regularized regression for face recognition with occlusion. Proceedings of the IEEE Conference on Computer Vision and Pattern Recognition Workshops.

[22] Wei X., Li C.T., Lei Z., Yi D., Li S.Z. (2014). Dynamic image-to-class warping for occluded face recognition. IEEE Trans. Inf. Forensics Secur..

[23] Xiong C., Zhao X., Tang D., Jayashree K., Yan S., Kim T.K. Conditional convolutional neural network for modality-aware face recognition. Proceedings of the IEEE International Conference on Computer Vision.

[24] Yu J., Lin Z., Yang J., Shen X., Lu X., Huang T.S. Generative image inpainting with contextual attention. Proceedings of the IEEE Conference on Computer Vision and Pattern Recognition.

[25] Mathai J., Masi I., AbdAlmageed W. (2019). Does generative face completion help face recognition?. Proceedings of the 2019 International Conference on Biometrics (ICB).

[26] Li H., Wang W., Yu C., Zhang S. (2021). SwapInpaint: Identity-specific face inpainting with identity swapping. IEEE Trans. Circuits Syst. Video Technol..

[27] Li L., Bao J., Yang H., Chen D., Wen F. (2019). Faceshifter: Towards high fidelity and occlusion aware face swapping. arXiv.

[28] Park T., Liu M.Y., Wang T.C., Zhu J.Y. Semantic image synthesis with spatially-adaptive normalization. Proceedings of the IEEE/CVF Conference on Computer Vision and Pattern Recognition.

[29] Liu H., Wan Z., Huang W., Song Y., Han X., Liao J. Pd-gan: Probabilistic diverse gan for image inpainting. Proceedings of the IEEE/CVF Conference on Computer Vision and Pattern Recognition.

[30] Li J., Li Z., Cao J., Song X., He R. FaceInpainter: High Fidelity Face Adaptation to Heterogeneous Domains. Proceedings of the IEEE/CVF Conference on Computer Vision and Pattern Recognition.

[31] Yeh R.A., Chen C., Yian Lim T., Schwing A.G., Hasegawa-Johnson M., Do M.N. Semantic image inpainting with deep generative models. Proceedings of the IEEE Conference on Computer Vision and Pattern Recognition.

[32] Yi D., Lei Z., Liao S., Li S.Z. (2014). Learning face representation from scratch. arXiv.

[33] Huang G.B., Mattar M., Berg T., Learned-Miller E. Labeled faces in the wild: A database forstudying face recognition in unconstrained environments. Proceedings of the Workshop on Faces in ‘Real-Life’ Images: Detection, Alignment, and Recognition.

[34] Liu Z., Luo P., Wang X., Tang X. Deep learning face attributes in the wild. Proceedings of the IEEE International Conference on Computer Vision.

[35] Ng H.W., Winkler S. (2014). A data-driven approach to cleaning large face datasets. Proceedings of the 2014 IEEE International Conference on Image Processing (ICIP).

[36] Van der Maaten L., Hinton G. (2008). Visualizing data using t-SNE. J. Mach. Learn. Res..

[37] Wang Z., Huang B., Wang G., Yi P., Jiang K. (2023). Masked face recognition dataset and application. IEEE Trans. Biom. Behav. Identity Sci..

[38] Ciampi L., Foszner P., Messina N., Staniszewski M., Gennaro C., Falchi F., Serao G., Cogiel M., Golba D., Szczęsna A. (2022). Bus violence: An open benchmark for video violence detection on public transport. Sensors.

[39] Foszner P., Szczęsna A., Ciampi L., Messina N., Cygan A., Bizoń B., Cogiel M., Golba D., Macioszek E., Staniszewski M. (2023). Crowdsim2: An open synthetic benchmark for object detectors. arXiv.

